# Exploring the effect and mechanism of Aloin A against cancer cachexia-induced muscle atrophy via network pharmacology, molecular docking, molecular dynamics and experimental validation

**DOI:** 10.18632/aging.205416

**Published:** 2023-12-29

**Authors:** Awaguli Dawuti, Lisha Ma, Xueyan An, Jiawei Guan, Changdong Zhou, Linyun He, Yue Xu, Bo Han, Abudumijiti Abulizi

**Affiliations:** 1 Key Laboratory of Xinjiang Phytomedicine Resource and Utilization, Ministry of Education, College of Pharmacy, Shihezi University, Shihezi 832002, China; 2 State Key Laboratory of Natural and Biomimetic Drugs, Peking University, Beijing 100191, China; 3 State Key Laboratory of Bioactive Substances and Functions of Natural Medicines, Institute of Materia Medica, Chinese Academy of Medical Sciences and Peking Union Medical College, Beijing 100050, China; 4 Division of Pharmaceutics and Pharmacology, College of Pharmacy, The Ohio State University, Columbus, OH 43210, USA

**Keywords:** Aloin A, cancer cachexia-induced muscle atrophy, network pharmacology, molecular docking, molecular dynamics, mice

## Abstract

80% of advanced cancer patients suffer from cachexia, but there are no FDA-approved drugs. Therefore, it is imperative to discover potential drugs.

Objective: This study aims at exploring the effect and targets of Aloin A against cancer cachexia (CC)-induced muscle atrophy.

Methods: Network pharmacology, molecular docking, molecular dynamics (MD) and animal model of CC-induced muscle atrophy with a series of behavior tests, muscle quality, HE staining and RT-PCR were performed to investigate the anticachectic effects and targets of Aloin A and its molecular mechanism.

Results: Based on network pharmacology, 51 potential targets of Aloin A on CC-induced muscle atrophy were found, and then 10 hub genes were predicted by the PPI network. Next, KEGG and GO enrichment analysis showed that the anticachectic effect of Aloin A is associated with PI3K-AKT, MAPK, TNF, TLR, etc., pathways, and biological processes like inflammation, apoptosis and cell proliferation. Molecular docking and MD results showed good binding ability between the Aloin A and key targets. Moreover, experiments *in vivo* demonstrated that Aloin A effectively rescued muscle function and wasting by improving muscle quality, mean CSA, and distribution of muscle fibers by regulating HSP90AA1/AKT signaling in tumor-bearing mice.

Conclusion: This study offers new insights for researchers to understand the effect and mechanism of Aloin A against CC using network pharmacology, molecular docking, MD and experimental validation, and Aloin A retards CC-induced muscle wasting through multiple targets and pathways, including HSP90AA1/AKT signaling, which provides evidence for Aloin A as a potential therapy for cancer cachexia in clinic.

## INTRODUCTION

Cancer cachexia is a complex metabolic disorder characterized by body weight loss, muscle wasting, and adipose tissue depletion during the treatment of some types of cancer, such as lung, colon, pancreas, etc., [1]. It is reported that the prevalence of cachexia is approximately 80% and cachexia not only highly influences patients’ quality of life and ability to tolerate treatment, but also reduced the survival [2, 3]. Indeed, it is showed that cachexia itself accounts for up to 30% of cancer-related mortality [4]. Anamorelin, a ghrelin receptor agonist, is the only clinically approved drug in Japan for cancer cachexia, but not approved by FDA because of the marginal effect on lean body mass and hand grip strength or patients’ quality of life [5]. Currently, there is no FDA-approved therapy that can effectively prevent and treat cancer cachexia [6]. Therefore, it is imperative to develop active compounds that are effective against cancer cachexia.

Traditional Chinese medicine (TCM) has been used for thousands of years in Asia, especially in China, Japan and Korea, and increasingly drawn the worldwide attention in recent years for its precise efficacy, relatively low cost and a few toxicity [7]. Recently, researchers have found that some TCM and its active ingredients have positive effects on cancer-related cachexia both in animal experiments [8–10] and clinical trials [11, 12], which are characterized by multiple targets and multiple pathways, suggesting that TCM may be an effective source for new drug development against cachexia. Aloin, a quality standard compound and bioactive components of *Aloe vera*, has a wide range of pharmacological activities, including anti-inflammatory, anti-tumor, anti-oxidative, anti-osteoporotic, anti-microbial, etc. indicating that Aloin has prominent healthcare and medical benefits and the potential to develop into promising health care products [13, 14]. Moreover, it is widely reported that these pharmacological activities may contribute to attenuating cancer cachexia and improving quality of life [1, 15–17]. However, there are no experimental or clinical studies on whether Aloin A has a therapeutic effect on cachexia.

Network pharmacology is one of the powerful bioinformatics tools that contributes to understanding the mechanism of TCM [18]. As an emerging comprehensive strategy, network pharmacology has offered a new direction for predicting potential therapeutic mechanisms of drugs by integrating multidirectional pharmaceutical biology, systems biology, bioinformatics, and computer science [19]. Through a universal analysis of the topological associations of “drug-target-disease”, network pharmacology offers a new perspective for modern research and the rational clinical use of TCM in a novel way.

**Figure 1:**
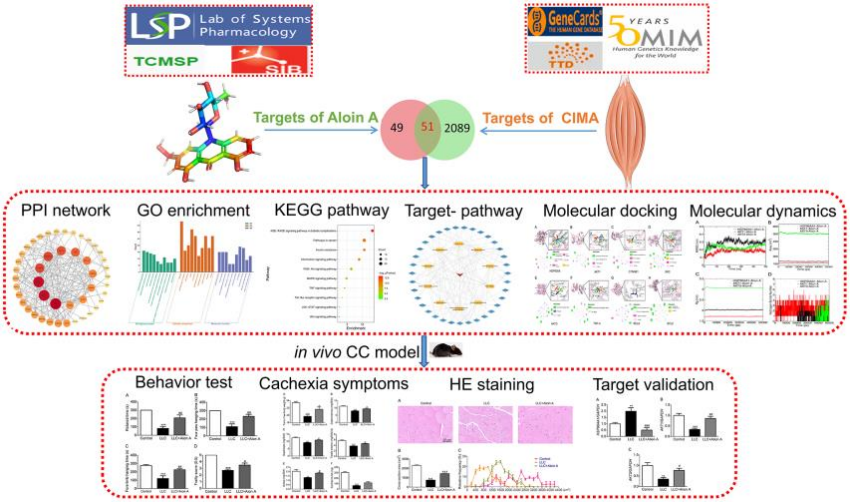
Flowchart of the network pharmacology, molecular docking, molecular dynamics and experimental validation to investigate pharmacological mechanism of Aloin A against CC-induced muscle atrophy.

In this study, we used systems pharmacology, molecular docking, and molecular dynamics-based strategy and experimental validation to explore the role and molecular mechanisms of Aloin A against cancer cachexia. Firstly, systems pharmacology was used to estimate the targets of Aloin A and targets of CC-induced muscle wasting by using several databases, then constructed PPI and predicted hub targets, and conducted Gene Ontology and KEGG pathway enrichment. Furthermore, the top targets were validated by molecular docking and molecular dynamics. Finally, we further validated the anti-cachectic effect of Aloin A in mice bearing lung tumors, and found that Aloin A retards CC-induced muscle atrophy through multiple targets and pathways, including HSP90AA1/AKT signaling. The Schematic diagram is illustrated in Figure 1.

## MATERIALS AND METHODS

### Systematic pharmacology and computational methods-based approach for the potential actions of Aloin A on CC-induced muscle atrophy

#### Potential target proteins of Aloin A and cancer related muscle atrophy

The chemical structure and computed properties of Aloin A (2D SMILES:C1=CC2=C(C(=C1)O)C(=O)C3=C (C2C4C(C(C(C(O4)CO)O)O)O)C=C(C=C3O)CO) were obtained from the PubChem database (https://pubchem.ncbi.nlm.nih.gov/, accessed on March 5, 2022). Potential targets of Aloin A (CAS: 1415-73-2) were retrieved from TCMSP (https://old.tcmsp-e.com/tcmsp.php, accessed on March 5, 2022) and SwissTargetPrediction (http://swisstargetprediction.ch/, accessed on March 8, 2022) databases. The target name was transformed into the gene symbol using the UniProt (https://www.uniprot.org/, accessed on March 9, 2022) database.

Additionally, disease targets for cancer-related muscle atrophy were collected from Genecards (https://www.genecards.org/, accessed on March 10, 2022), TTD (http://db.idrblab.net/ttd/, accessed on March 10, 2022), and DisGeNET (https://www.disgenet.org/home/, accessed on March 10, 2022), and duplicated disease targets were removed. Finally, we analyzed the target intersection between Aloin A and CC-induced muscle atrophy by Draw Venn Diagram.

### Construction of protein–protein interaction (PPI) network

PPI network was obtained from STRING database (https://string-db.org/, accessed on October 8, 2022). The species named “Homo sapiens”, and proteins corresponding to genes with high confidence were selected based on the minimum required interaction scores >0.7. The data were saved in TSV format and then imported into Cytoscape software (version 3.9.1) to visualize the PPI network.

### GO and KEGG pathway enrichment analysis

To explore the biological function of potential targets of Aloin A on cancer cachexia, Gene Ontology (GO) analysis, including biological processes (BP), cellular components (CC) and molecular functions (MF), and the KEGG pathway were integrated Discovery (DAVID) (https://david.ncifcrf.gov/, accessed on October 10, 2022) database and conducted using bioinformatics.

### Target identification based on the strategy of molecular docking

Three-dimensional (3D) structures of the key proteins and potential pocket were obtained from the RCSB PDB database (https://www.rcsb.org/, accessed on October 14, 2022) and DoGSiteScorer of Proteins Plus (https://proteins.plus/, accessed on October 14, 2022), respectively. Three-dimensional (3D) structure of Aloin A was downloaded from PubChem database (https://pubchem.ncbi.nlm.nih.gov/, accessed on October 14, 2022). Discovery Studio Visualizer 2019 has been used to hydrogenate proteins, remove water and ligand molecules. AutoDockTools ver. 1.5.7 was used to convert Aloin A and protein molecules into “pdbqt” format, and finally vina was used for molecular docking of HSP90AA1 (grid size: x = 0.847154, y = 29.73, z = 28.0159; box size: 58.392), AKT1 (grid size: x = 22.9599, y = −15.8276, z = −16.0423; box size: 72.98Å), CTNNB1 (grid size: x = 56.7304, y = 19.0907, z = 23.0016; box size: 93.012 Å), SRC (grid size: x = −10.4861, y = 45.1305, z = 23.0835 ; box size: 70 Å), AKT3 (grid size: x = 29.6112, y = 29.6112, z = −1.14428; box size: 80Å), TNF-α (grid size: x = 21.4268, y = 17.9164, z = 21.4864; box size: 76.992Å), RELA (grid size: x = 9.13265, y = 6.74423, z = 1.06598; box size: 124.31Å), BCL2L1 (grid size: x = 31.7336, y = 4.86445, z = 26.3051; box size: 110Å) with Aloin A. Ligand-macromolecule complexes with lower binding energy were regarded as more favorable potential target proteins and we selected binding energy less than −7.0 kcal/mol. The compound–target interactions and modes of binding were visualized using PyMoL and Discovery Studio 2019.

### Target identification based on the strategy of molecular dynamic (MD) simulation

The related parameters of MD may better investigate the stability and conformational changes of a protein binding to the ligand and can be used to further explore its mechanism of action [20]. GROMACS 2021.5 software package was used to simulate the dynamics of the three systems. Proteins used OPLS-AA/L force field, and small molecular ligands were prepared with the LigParGen server (https://traken.chem.yale.edu/ligpargen). Each simulation system was placed in A SPCE water box model with a side length of 10 Å and randomly added sodium ions or chloride ions to maintain the system’s electrical neutrality. After the system was built, the energy optimization process was performed using the steepest descent method. Next, NVT and NPT balances of 1000 ps were performed, respectively. MD simulation production of each protein ligand complex runs up to 50 ns. Consequently, various geometric properties of the system, such as root mean square deviation (RMSD), root mean square fluctuation (RMSF), solvent accessible surface area (SASA), and hydrogen bond number, are calculated according to the trajectory for further analysis. The binding free energies (ΔGbind) including electrostatic interactions (ΔEelec), Vander Waals interactions (ΔEvdW), non-polar solvation energy (ΔGSASA) and polar solvation energy (ΔGpolar) were calculated using the molecular mechanics Poisson–Boltzmann surface area (MM-PBSA) method implemented in GROMACS compatible tool “gmx_mmpbsa”, and the trajectory of the last 10 ns is selected for MM_PBSA calculation.

### *In vivo* experiments to validate the anti-cachectic effect of Aloin A in mice bearing lung tumor

#### Reagents and animals

Aloin A was purchased from Wuhan ChemFaces Biochemical Co., Ltd., (purity >98%). 8-week-old C57BL/6 male mice (*n* = 8/group) were purchased from Skbex Biotechnology, Henan, China. The experiments were performed according to the National Institutes of Health Guidelines on the Use of Laboratory Animals. The University Animal Care Committee for Animal Research of Shihezi University (Approval Number: A2022-090-01, 2022) approved the study protocol (Xinjiang, China).

### Tumor cell culture

Lung cancer cells (LLC) were provided by the Cell Resource Center, Institute of Basic Medicine, Chinese Academy of Medical Sciences (Beijing, China). The cells were cultured in Dulbecco’s Modified Eagle Medium (Biological Industries, Beit-Haemek, Israel) supplemented with 10% FBS (Biological Industries, Beit-Haemek, Israel) and 1% penicillin-streptomycin solution in digital incubator with 5% CO2 at 37°C. The cells were resuspended in phosphate-buffered saline (PBS) for establishment of cancer cachexia induced muscle atrophy animal model.

### Model of cancer cachexia and drug treatment

For the establishment of cancer cachexia model, LLC cells (1 × 106 in 100 μL) or an equal volume of PBS was injected subcutaneously into the flanks of C57BL/6 mice as described previously [21]. One week after tumor inoculation, experimental mice were divided into three groups: control group, LLC model group, and LLC+Aloin A group. LLC+Aloin A group mice were orally administrated Aloin A (20 mg/kg/day) [22–24] every day for 16 days. The control and LLC group mice received the same volume of saline.

### Rotarod test

Muscle function of tumor-bearing mice was evaluated by rotarod apparatus (ZB200-Taimeng, Chengdu, China) as previously reported [25]. On day 13, after acclimation for half an hour in the testing room, the mice (*n* = 8) were trained on a rotating rod (2–20 rpm) for 5 min. During habituation, the mice were immediately placed back on the rod if they fell off. After 0.5 h, muscle function was evaluated (3 trials, interval of 15 min) as the speed was accelerated from 5–30 rpm and latency to fall was recorded.

### Inverted screen test

Muscle strength was evaluated using an inverted screen test [26]. Briefly, on day 14, the mice (*n* = 8) were placed on a wire mesh screen, and the screen was inverted. Muscle strength was calculated by measuring the hanging duration.

### Wire grip test

The muscle strength of the mice was evaluated as previously described [27]. On day 15, all mice (*n* = 8) were allowed to grasp a metal wire (2 mm diameter), and the grasp of time was recorded until the mice fell off. The test score was calculated as the mean of at least three repetitions. Scores were assigned as follows: 0 points: fell off the wire within 10 seconds; 1 point: lifted one of the hind limbs; 2 points: tried to climb the wire; 3 points: grasped the wire with the front claw and at least one rear claw; 4 points: wrapped the legs and tail around the wire; and 5 points: tried to escape to the end of the wire.

### Histological staining

Skeletal muscles (SM, *n* = 3) were minced, fixed in 10% formalin in 0.01 M PBS, dehydrated, and embedded in paraffin. 5 μm-thick slices of SM were cut. After deparaffinization, SM sections were rehydrated, stained with hematoxylin and eosin (H&E), and the slides were viewed, and photomicrographs were captured under a light microscope.

### Real-time PCR

Real-time PCR (RT-PCR) was performed as described previously [28]. According to the manufacturer’s instructions, total RNA was extracted from muscle tissue (*n* = 6) with TRIzol (Sangon Biotech, Shanghai, China) and cDNA was prepared by using RevertAid First Strand cDNA Synthesis Kit (Thermo Fisher Scientific, Waltham, MA, USA). RT-qPCR was performed using StepOnePlus™ RT-PCR System (Qiagen, Hilden, Germany) with SYBR green (Qiagen, Hilden, Germany) and appropriate primers, and specific primer sequences are listed in Table 1.

**Table 1:** Primer sequences used in RT-qPCR.

**Gene**		**Sequence (5′→3′)**
*HSP90AA1*	F	ACGAAGCATAACGACGATGAGCAG
	R	CATTGGTTCACCTGTGTCAGTCCTC
*AKT1*	F	AGCGACGTGGCTATTGTGAAG
	R	GCCATCATTCTTGAGGAGGAAGT
*AKT3*	F	AGGGCTGTCTGGGAGGAAACTG
	R	TGGAGGTCAGGTGGGAAATGGG
*GAPDH*	F	GGGTCCCAGCTTAGGTTCAT
	R	CCCAATACGGCCAAATCCGT

### Statistical analysis

The statistical data were analyzed with GraphPad Prism software (GraphPad Software, Inc., San Diego, CA, USA). One-way analysis of variance (ANOVA) was used to compare the differences between each group. *P* < 0.05 was considered statistically significant. All data are expressed as the mean ± SEM.

### Data availability statement

All data generated or analyzed during this study are included in this article and are available from the corresponding author on reasonable request.

## RESULTS

### Network pharmacology analysis

#### Target prediction of Aloin A in cancer related muscle atrophy

**Figure 2:**
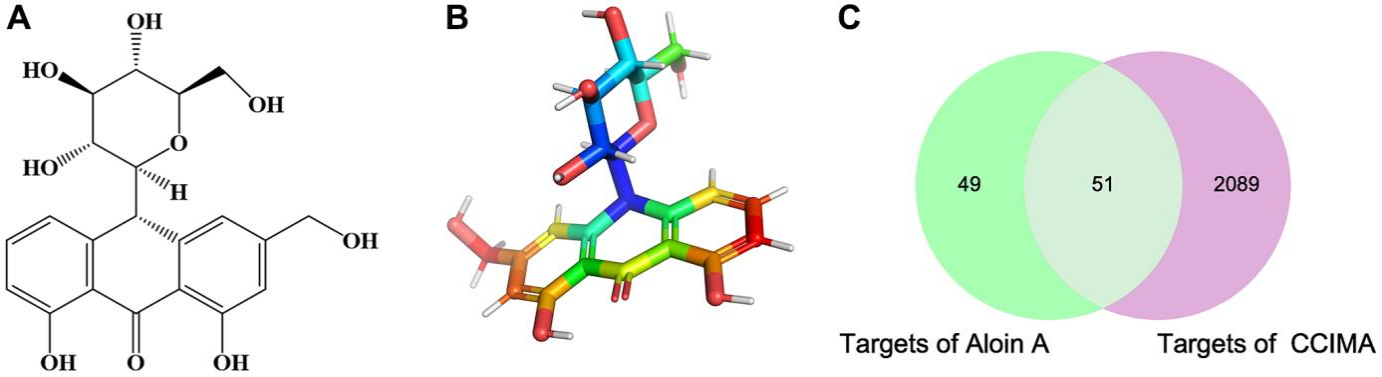
**The chemical structure of Aloin A and target prediction of Aloin A in cancer related muscle atrophy.** (**A**) 2 D structure of Aloin A. (**B**) 3 D structure of Aloin A. (**C**) Wayne diagram of common targets of Aloin A (drug) and cancer related muscle atrophy (disease).

The chemical structure and computed properties of Aloin A were obtained from the PubChem database (Figure 2A, 2B). Based on TCMSP and SwissTargetPrediction databases, 100 potential targets of Aloin A were identified. In addition, 2140 potential targets of cancer related muscle atrophy were obtained based on the DrugBank, TTD and OMIM databases. Finally, the 100 compound targets and 2140 disease targets have 51 overlaps (Figure 2C), suggesting that these 51 genes may be key targets for the treatment cachexia induced muscle wasting with Aloin A.

### PPI network analysis

**Figure 3:**
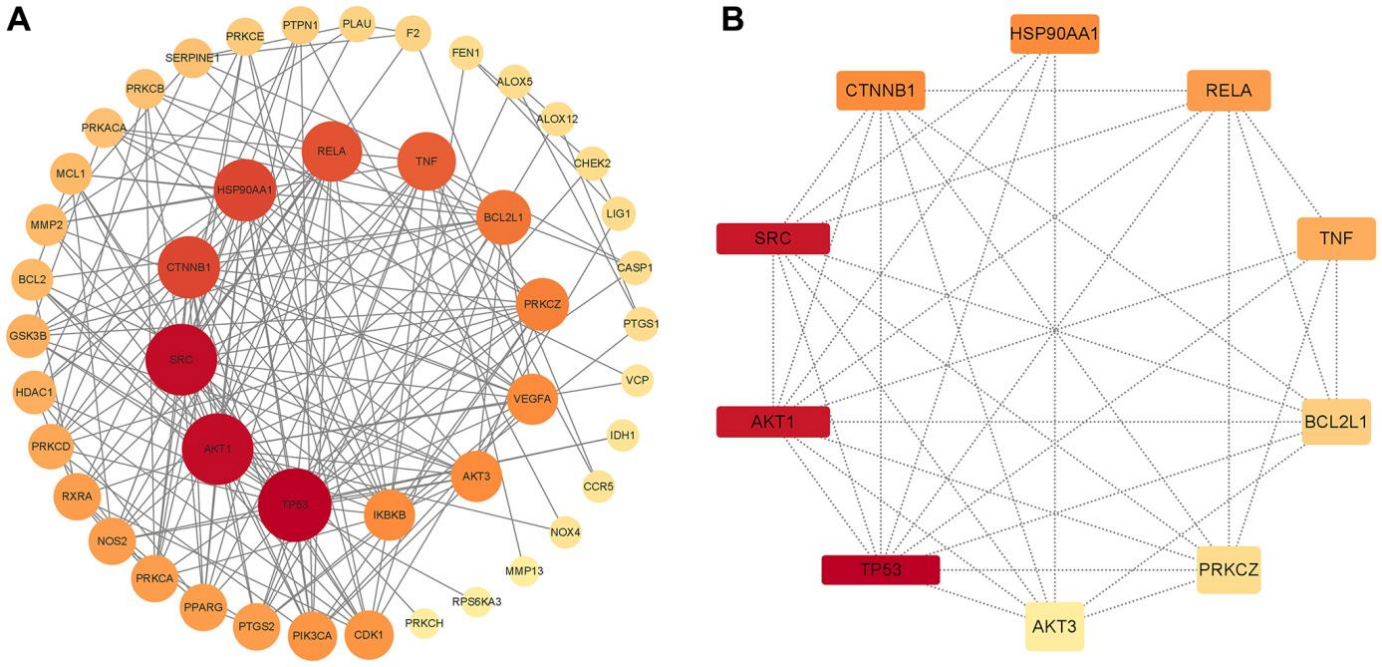
**PPI network of common compound–disease targets and hub genes.** (**A**) PPI network of common compound–disease target. (**B**) The hub 10 genes. The size and color of nodes indicate the magnitude of the degree. A larger size of a node means a larger degree.

To further clarify the correlations between Aloin A targets associated with cancer related muscle atrophy targets, a “target-target network” was built based on STRING results from 51 overlapping genes with high confidence (>0.7). Edges and nodes represent targets and interactions between targets in the PPI network, respectively. The color and size of the nodes demonstrate the magnitude of the degree. A larger sized node means a larger degree. A total of 52 nodes and 195 edges were involved in the PPI network, as shown in Figure 3A. The hub 10 targets included TP53, AKT1, SRC, CTNNB1, HSP90AA1, RELA, TNF, BCL2L1, PRKCZ and AKT3 (Figure 3B and Table 2).

**Table 2:** The top 10 hub genes with higher degree of connectivity.

**Gene ID**	**Gene name**	**Gene symbol**	**Degree**
7157	tumor protein p53	TP53	24
6714	SRC proto-oncogene, non-receptor tyrosine kinase	SRC	23
207	AKT serine/threonine kinase 1	AKT1	23
1499	catenin beta 1	CTNNB1	18
3320	heat shock protein 90 alpha family class A member 1	HSP90AA1	18
5970	RELA proto-oncogene, NF-kB subunit	RELA	17
7124	tumor necrosis factor	TNF	16
598	BCL2 like 1	BCL2L1	14
5590	protein kinase C zeta	PRKCZ	13
10000	AKT serine/threonine kinase 3	AKT3	12

### Gene ontology enrichment analysis

**Figure 4:**
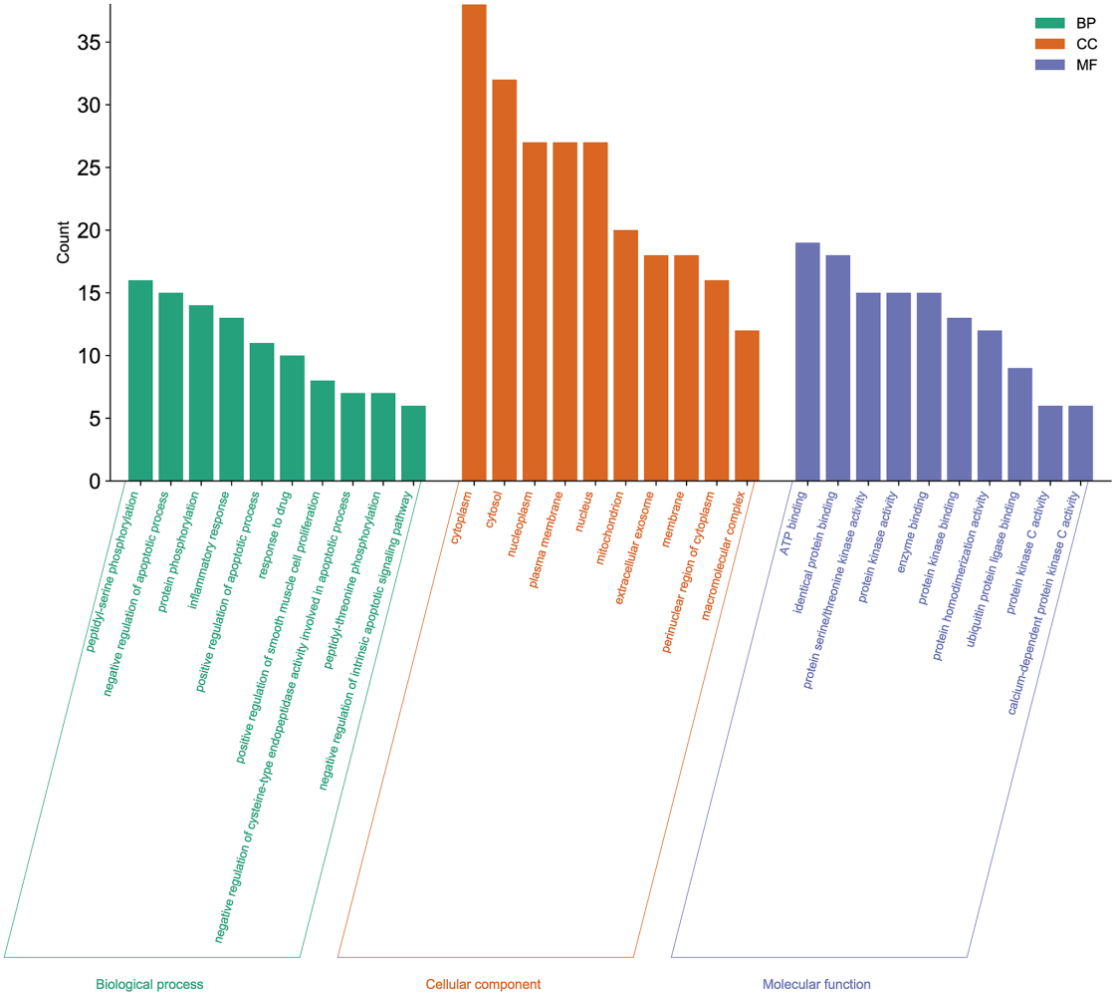
**Gene Ontology enrichment analysis of the potential targets of Aloin A against cancer related muscle atrophy.** Top 10 biological process (BP) terms, cellular component (CC) terms, and molecular function (MF) terms are shown as green, orange, and purple bars, respectively.

For the 51 genes corresponding to potential therapeutic targets, we constructed a Gene Ontology (GO) enrichment analysis to identify relevant biological processes, cellular components, and molecular functions (Figure 4). We choose the top 10 terms for a brief demonstration. The results showed that a series of biological processes are involved in the treatment of cancer related muscle atrophy, including peptidyl-serine phosphorylation, negative regulation of apoptotic process, protein phosphorylation, inflammatory response, positive regulation of apoptotic process, positive regulation of smooth muscle cell proliferation, negative regulation of intrinsic apoptotic signaling pathway, and others.

### KEGG pathway enrichment analysis and target-pathway network analysis

**Figure 5:**
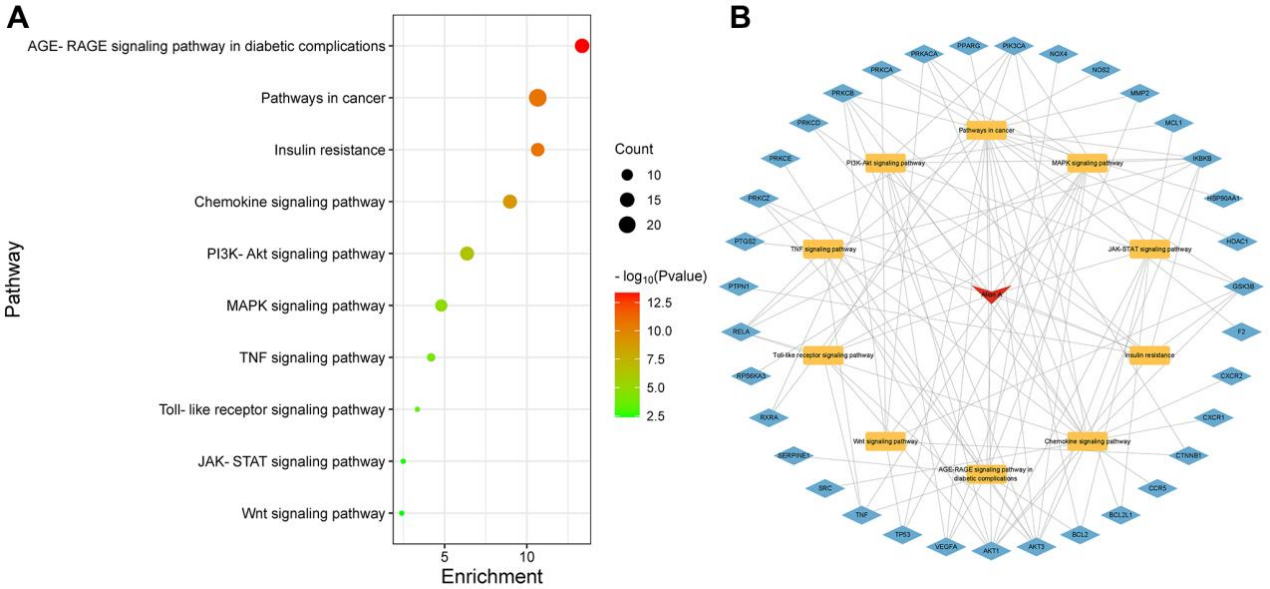
**Kyoto Encyclopedia of Genes and Genomes (KEGG) enrichment analysis.** (**A**) KEGG enrichment analysis. The Y-axis represents KEGG pathways. The X-axis indicates the enrichment *p*-value. (**B**) Drug-Pathway-Target construction. The red represents drug. The yellow represents related pathway. The blue represents related target genes.

KEGG pathway enrichment analysis was performed, as shown in Figure 5A. The Y-axis indicates the KEGG pathways and the X-axis represents the *p*-value. The redder the color, and the smaller the value of *p* means more credibility and more importance. In this study, we selected the key 10 pathways from small to large according to the *p*-value for a brief demonstration. Among these pathways, the AGE-RAGE signaling pathway in diabetic complications, insulin resistance, chemokine signaling pathway, PI3K-Akt signaling pathway, MAPK signaling pathway, TNF signaling pathway, TLR signaling pathway, JAK-STAT signaling pathway and Wnt signaling pathway are considered the top priority. The top 10 signaling pathways with associated genes were mapped using Cytoscape3.9.0 software as shown in Figure 5B, indicating that Aloin A could alleviate cancer cachexia-induced muscle wasting by regulating multiple signaling pathways through multiple genes.

### Molecular docking validation of Aloin A–key target interactions

**Figure 6:**
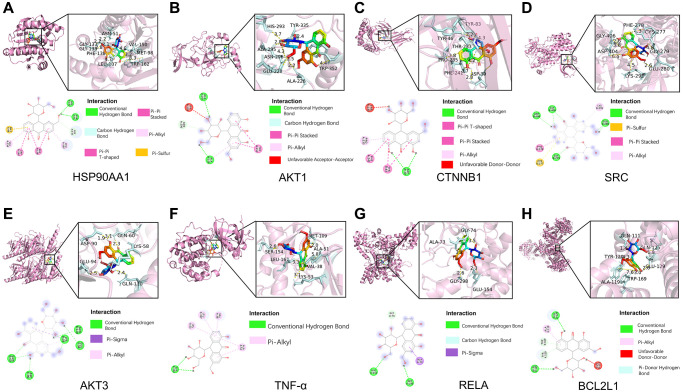
**Molecular docking of Aloin A with top targets.** (**A**) Aloin A-HSP90AA1. (**B**) Aloin A-Akt1. (**C**) Aloin A-CTNNB1. (**D**) Aloin A-SRC. (**E**) Aloin A-AKT3. (**F**) Aloin A-TNF-α. (**G**) Aloin A-RELA. (**H**) Aloin A-BCL2.

Next, for validating the results of systematic pharmacology, we conducted molecular docking to evaluate the screened key targets. Based on the degrees of key targets in the PPI network and the KEGG results, Aloin A were analyzed by molecular docking with the hub genes HSP90AA1, AKT1, CTNNB1, SRC, AKT3, TNF-α, RELA, BCL2L1, respectively. The molecular docking results demonstrated that the binding energies (Vina scores) of Aloin A with the above key targets were in the range of −7.9 to −8.9 kcal/mol, (Table 3 and Figure 6A–6H), suggesting that Aloin A stably binds to those targets.

**Table 3:** The binding energy of molecular docking.

**Target**	**Compound**	**Target (PDB ID)**	**Binding energy (kcal/mol)**
HSP90AA1	Alion A	6ltk	−8.9
AKT1		3os5	−8.8
CTNNB1		6o9c	−8.7
SRC		4u5j	−8.5
AKT3		2x18	−8.3
TNF-α		3mw1	−8.3
RELA		3rc0	−8.3
BCL2L1		6uvf	−7.9

The bind energy of Aloin A with HSP90AA1 was −8.9 kcal/mol, and the binding affinity was contributed by the following: The conventional hydrogen bond formed with GLY137 and ASN51 residues; Pi-pi stacked bond formed with TRP162 residues; Carbon-hydrogen bond formed with GLY135 residues; The hydrophobic interaction (π-Alkyl) formed with LEU107 and VAL150 residues; Pi-pi T-shaped bond formed with PHE138 residues; Pi-sulfur bond formed with MET98 residues (Figure 6A).

The bind energy of Aloin A with AKT1 was −8.8 kcal/mol, and the binding affinity was contributed by the following: The conventional hydrogen bond formed with ALA226 and ALA295 residues; Carbon-hydrogen bond formed with ASN296 residues; Pi-pi stacked bond formed with TRP352 residues; The hydrophobic interaction (π-Alkyl) formed with ALA226 residues; Unfavorable Acceptor-Acceptor bond formed with HIS293 residues (Figure 6B).

The bind energy of Aloin A with CTNBB1 was −8.7 kcal/mol, and the binding affinity of was contributed by the following: The conventional hydrogen bond formed with ASP30 residues; Pi-pi T-shaped bond formed with PHE241 residues; Pi-pi stacked bond formed with TYR83 residues; The hydrophobic interaction (π-Alkyl) formed with PRO235 residues; Unfavorable Donor-Donor bond formed with THR233 residues (Figure 6C).

The bind energy of Aloin A with SRC was −8.5 kcal/mol, and the binding affinity was contributed by the following: The conventional hydrogen bond formed with ASP404, GLY406, GLY279 and GLU280 residues; Pi-sulfur bond formed with CYS277 residues; Pi-pi stacked bond formed with PHE278 residues; The hydrophobic interaction (π-Alkyl) formed with LYS295 residues (Figure 6D).

The bind energy of Aloin A with AKT3 was −8.3 kcal/mol, and the binding affinity was contributed by the following: The conventional hydrogen bond formed with GLU110, GLU94, GLN60, LYS58 and ASP90 residues; Pi-sigma bond formed with LYS58 residues; The hydrophobic interaction (π-Alkyl) formed with LYS58 residues (Figure 6E).

The bind energy of Aloin A with TNF-α was −8.3 kcal/mol, and the binding affinity was contributed by the following: The conventional hydrogen bond formed with SER154 residues; The hydrophobic interaction (π-Alkyl) formed with VAL38, LEU167, LYS53 and ALA51 residues (Figure 6F).

The bind energy of Aloin A with RELA was −8.3 kcal/mol, and the binding affinity was contributed by the following: The conventional hydrogen bond formed with GLU154 and GLY298 residues; Carbon Hydrogen Bond formed with GLY74 residues; The Pi-Sigma formed with ALA73 residues (Figure 6G).

The bind energy of Aloin A with BCL2L1 was −7.9 kcal/mol, and the binding affinity was contributed by the following: The conventional hydrogen bond formed with GLU129 and GLN125 residues; Pi-Alkyl bond formed with ALA119 residues; Unfavorable Donor-Donor bond formed with GLN111 residues; Pi-Donor Hydrogen bond formed with TRP169 and TYR120 residues;(Figure 6H). The above results implying that Aloin A may be exert anti-cachexia effect by interacting with these residues for stabling the key targets.

### Validation of Aloin A-target interactions by molecular dynamics simulation

**Figure 7:**
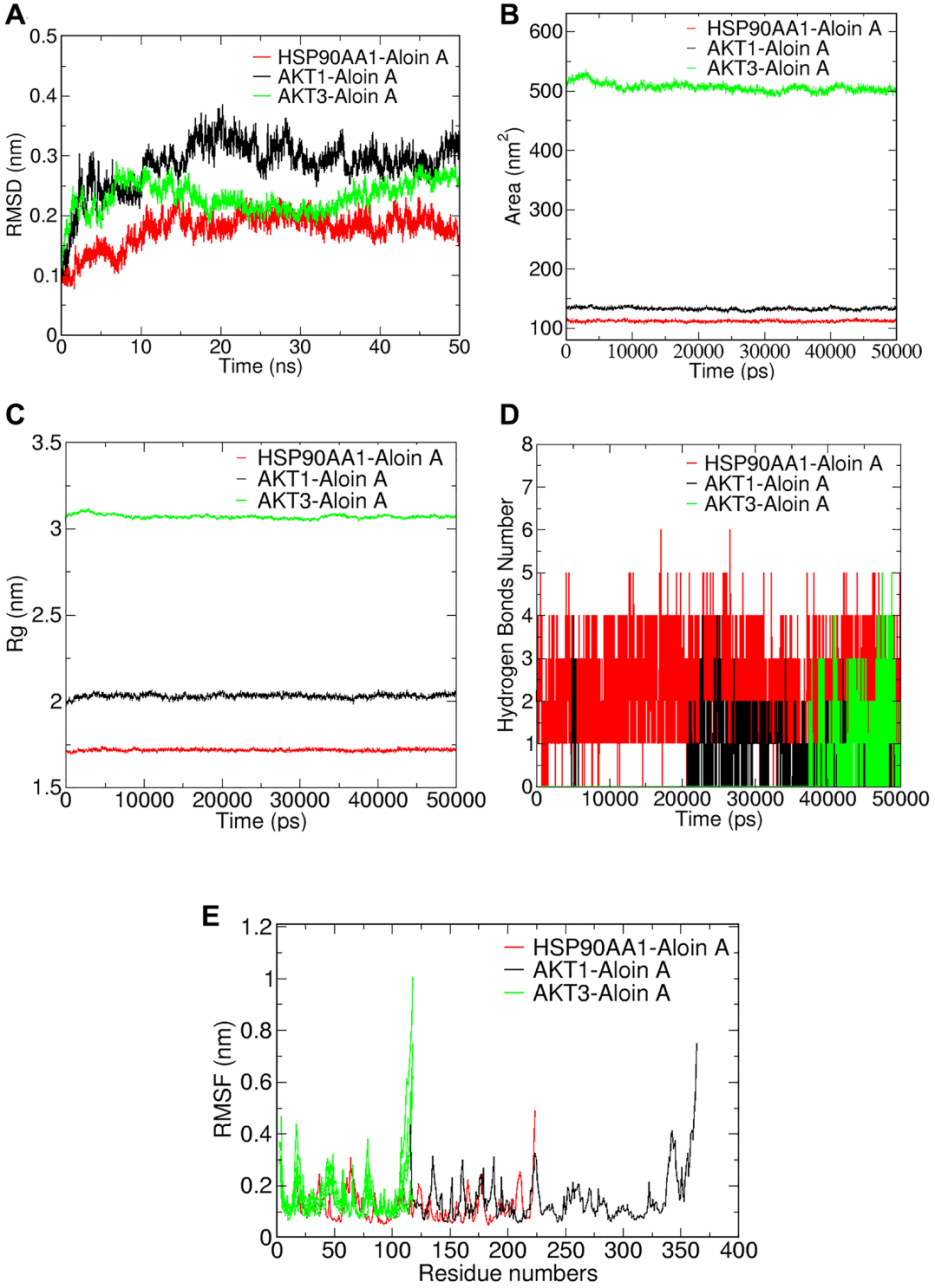
**Validation of Aloin A-target interactions by molecular dynamics simulation.** (**A**) Root mean square deviations (RMSD) values. (**B**) Solvent-accessible surface area (SASA) area analysis. (**C**) Radius of gyration (Rg) analysis. (**D**) Hydrogen bond analysis. (**E**) Root mean square fluctuations (RMSF) values during molecular dynamics simulations.

To better understand protein-ligand interactions, we selected representative molecular docking complexes for MD simulation and analysis. To investigate the stability of Aloin A binding to selected proteins, we calculated the RMSD of the skeleton atoms in the simulated trajectory. RMSD has been commonly used to estimate the degree of protein deviation from the original structure, which explains the stability of the complex [29]. As shown in Figure 7A, the plot of the HSP90AA1-Aloin A complex showed a stable equilibrium approximately after 15 ns, then a stable RMSD could be seen approximately at ~0.20 nm. The plot of AKT1-Aloin A complex showed a stable equilibrium approximately after 25 ns, then a stable RMSD could be seen approximately at ~0.3 nm. The RMSD value of the AKT3-Aloin A complex stabilized at about 0.2–0.28 nm between 18 and 50 ns. In general, the RMSD of the above three complexes remains stable after 15 ns. Among them, the stability of HSP90AA1-Aloin A complex is the best.

In addition, we analyzed the solvent accessible surface area (SASA) of the complexes by simulating the trajectory to evaluate the volume change of the complex. As shown in Figure 7B, the SASA of AKT1-Aloin A and HSP90AA1 complexes are similar and fluctuate less than the AKT3-Aloin A complex during entire simulation, indicating that although the SASA of three complexes is stable throughout the whole simulation process, the stability of AKT1-Aloin A and HSP90AA1 complexes is less affected by solvent and has better stability in aqueous solution.

The radius of gyration (Rg) reflects the binding tightness and degree of constraint of the system, and a higher Rg value is associated with a better chance of producing flexible ligands. Therefore, the higher the Rg value, the lower the stability [30]. As shown in Figure 7C, HSP90AA1-AloinA complex has the smallest Rg below 1.75 and is the most stable system compared to the Rg of AKT1-AloinA complex and AKT3-AloinA complex, their values fluctuate around 2.1 nm and 3.1 nm, respectively. In general, the Rg of the above three complexes is stable at 0–50 ns.

Hydrogen bond is one of the important parameters to reflect the stability of protein and ligand binding. Therefore, we further studied the variation of the number of hydrogen bonds in the complex during the MD simulation. As shown in Figure 7D, we found there was a maximum occupancy of 6 hydrogen bonds between HSP90AA1 and Aloin A, and the number of hydrogen bonds was stable between 1 and 4 during the MD simulation of 0–50 ns. The number of hydrogen bonds mostly concentrated between 1 and 3 for the complex of AKT1-Aloin A and the complex of AKT3-Aloin A.

RMSF mainly reflects the flexibility of residues of amino acids in the protein, which is important to deeply understanding the local conformational changes of protein side chains in MD simulated time [29]. The greater the fluctuation of RMSF of the system, the higher the flexibility of amino acids, resulting in unstable binding [31]. We further performed RMSF analysis to assess the positional fluctuation of each amino acid around its average position and stability of system. As shown in Figure 7E, there are large fluctuations in the protein terminal residues of the three complexes during the simulation process, it has little effect on the stability of the complexes because it is not the active site of the protein. Luckily, we found that there was little fluctuation in active sites of the protein in each complex. HSP90AA1-AloinA complexes fluctuated less at residues 101–107, 117–140, and AKT1-AloinA complexes fluctuated less at residues 122–128, 130–139, respectively, and AKT3-Aloin A complex fluctuated less at residues 77–78. In summary, the flexibility of these three compounds is low and has a good stability.

### The analysis of Aloin A-target interactions by binding free energies

**Figure 8:**
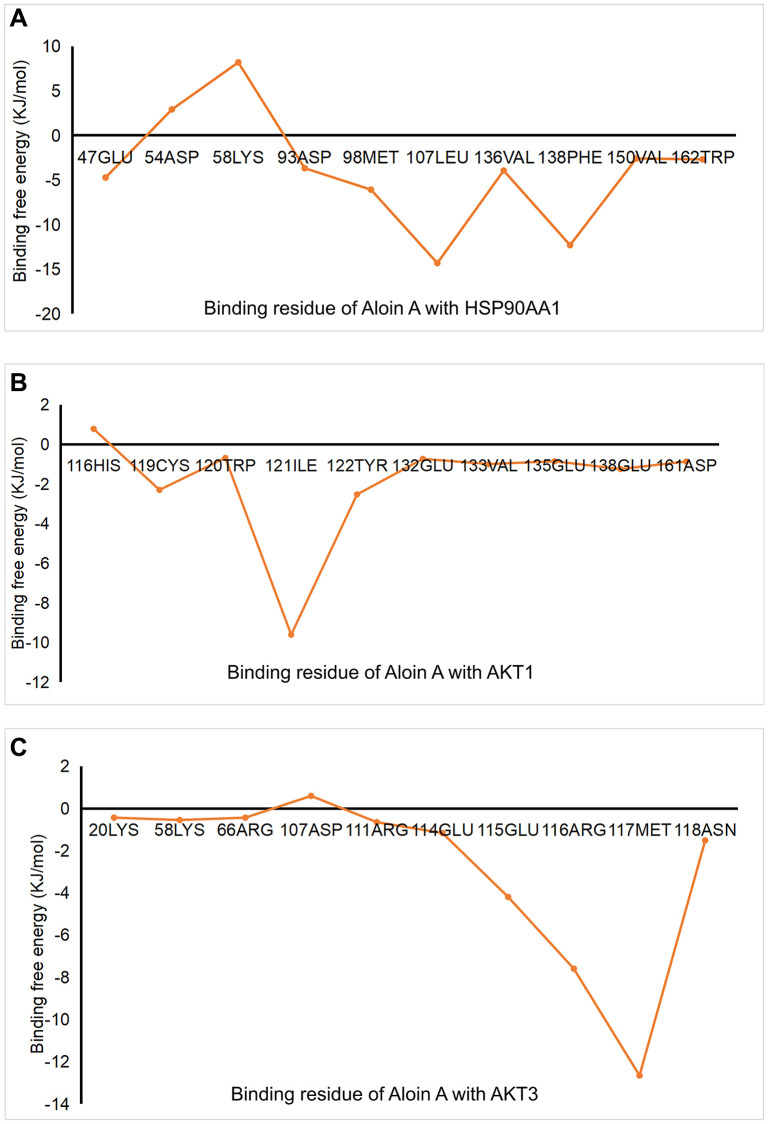
**The analysis of Aloin A-target interactions by binding free energies.** (**A**) Binding free energy of HSP90AA1-Aloin A. (**B**) Binding free energy of AKT-Aloin A. (**C**) Binding free energy of AKT3-Aloin A.

In order to better understand intermolecular interactions and the stability of complexes, we conduct the analysis of the binding free energy. The results showed that all binding free energy was less than zero, indicating that the reaction can proceed spontaneously (Table 4). The binding free energies of AKT3-Aloin A complex only has a van der Waals energy and electrostatic energy, but the other two complexes were decomposed into four energy components: Van der Waals energy, electrostatic energy, polar solvation energy and SASA non-polar solvation energy to get insights into their individual contributions. Among them, van der Waals force plays an important role in the binding process in this study. HSP90AA1-Aloin A complex displayed portrayed highest free energy (–79.450 kJ/mol), whereas AKT1-Aloin A complex possesses the least negative binding energy (−38.544 kJ/mol) (Table 4). To analyze the contribution of residues to protein ligand interaction, free energy decomposition per residue was employed (Figure 8). The results showed that MET-98, PHE-138 and LEU-107 make the greatest contribution to binding free energy for the HSP90AA1 system (Figure 8A). CYS-119, ILE-121 and TYR-122 make the greatest contribution to the binding free energy for the AKT1 system (Figure 8B), while GLU-115, ARG-116 and MET-117 contribute most to that of AKT3 system (Figure 8C).

**Table 4:** The binding free energy of each complex and various energy components.

**Complexes**	**ΔE_vdw_ (kJ/mol)**	**ΔE_elec_ (kJ/mol)**	**ΔG_polar_ (kJ/mol)**	**ΔG_SASA_ (kJ/mol)**	**ΔG_bind_ (kJ/mol)**
HSP90AA1-Aloin A	−191.749	−45.640	183.540	−25.601	−79.450
AKT3-Aloin A	−51.708	−6.184	0.000	0.000	−57.892
AKT1-Aloin A	−58.176	−5.453	34.024	−8.938	−38.544

### Effect of Aloin A against cachexia-induced muscle atrophy in mice bearing lung tumor

#### Effect of Aloin A on muscle dysfunctional behavior in mice bearing lung tumor

**Figure 9:**
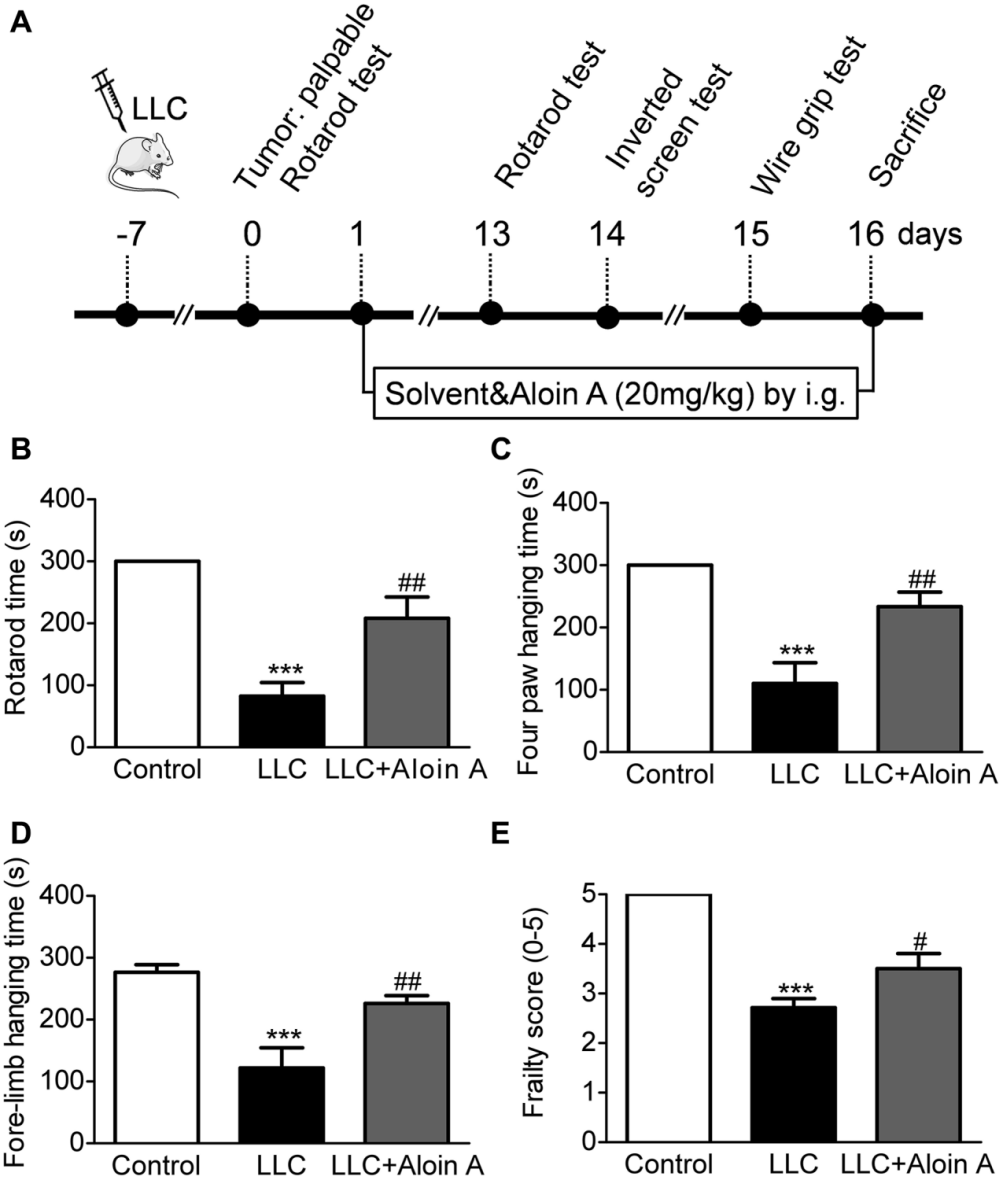
**Effect of Aloin A on muscle dysfunctional behavior in mice bearing lung tumor.** (**A**) Study design. (**B**) Rotarod time on rotarod test. (**C**) Four paws hanging time on inverted screen test. (**D**) Fore-limb hanging time on wire grip test. (**E**) Frailty score on wire grip test. The values are shown as the mean ± SEM (*n* = 8). ^***^P < 0.001 vs. the control group. ^#^*P* < 0.05, ^##^*P* < 0.01 vs. the LLC group.

As shown in Figure 9A, the mice were subjected to behavioral tests before sacrifice, the results of the rotarod test, the wire grip test and the inverted screen test are shown in Figure 9. Compared to the control group, rotary time (Figure 9B), four paws hanging time (Figure 9C), fore-limb hanging time (Figure 9D) and frailty score (Figure 9E) were significantly decreased in LLC group, and Aloin A (20 mg/kg) treatment improved these abnormal behaviors in LLC group mice, indicating that Aloin A can effectively alleviate muscle function in mice bearing lung tumor.

### Effect of Aloin A on body weight, muscle quality and adipose mass in mice bearing lung tumor

**Figure 10:**
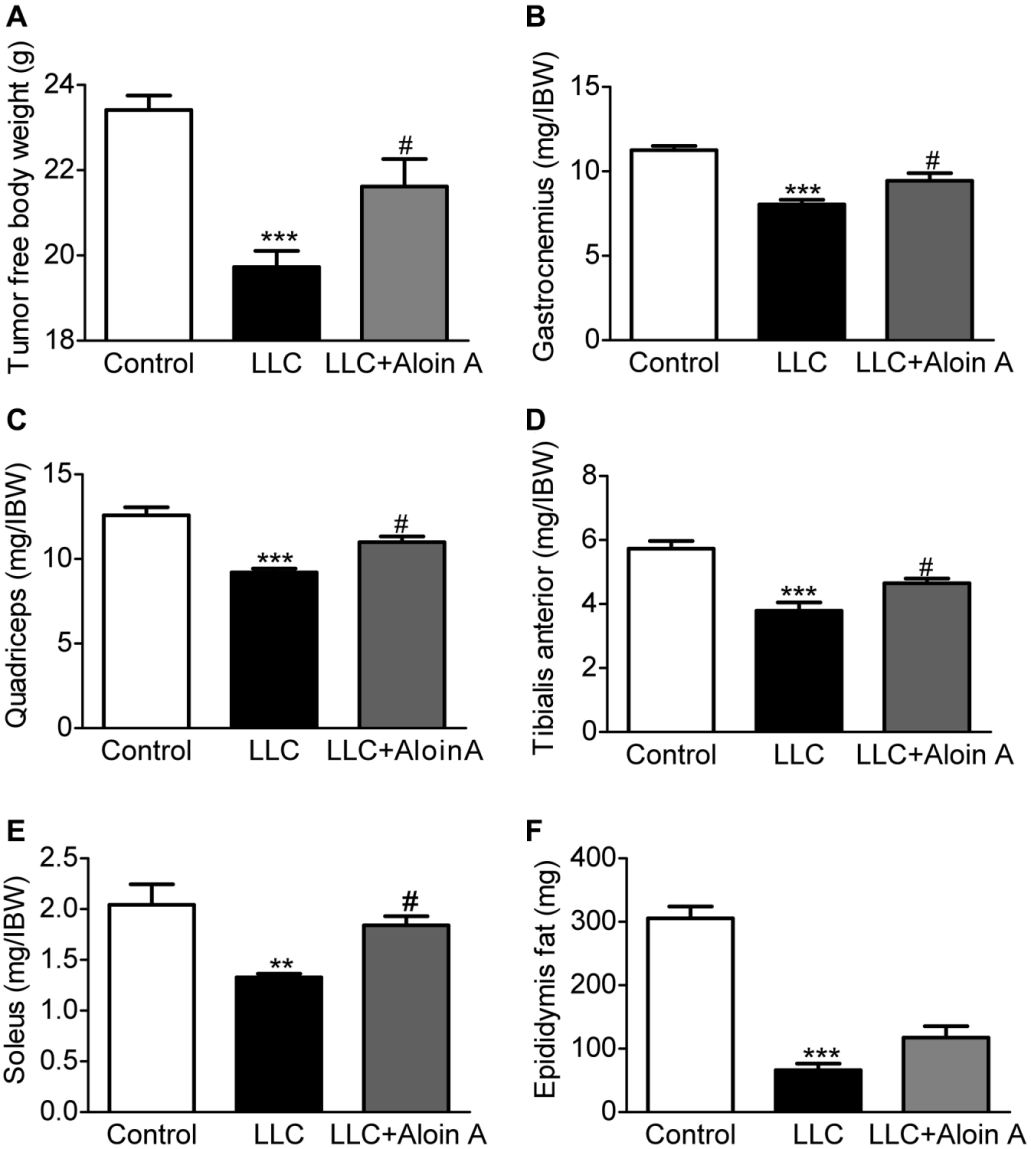
**Effect of Aloin A on body weight, muscle quality and adipose mass in mice bearing lung tumor.** (**A**) The tumor free body weight to initial body weight (IBW) ratio. (**B**) Gastrocnemius muscle to initial body weight (IBW) ratio. (**C**) Quadriceps to initial body weight (IBW) ratio. (**D**) Tibialis anterior muscle to initial body weight (IBW) ratio. (**E**) Soleus to initial body weight (IBW) ratio. (**F**) Epididymis fat mass. The values are shown as the mean ± SEM (*n* = 8). ^**^P < 0.01, ^***^P < 0.001 vs. the control group. ^#^*P* < 0.05 vs. the LLC group.

It is well known that cachexia is a complex metabolic disorder characterized by body weight loss, muscle wasting, and adipose tissue depletion. Therefore, we measured tumor free body weight, muscle mass and epididymis fat mass, and found that compared with the control group, tumor free body weight (Figure 10A), muscle masses, including gastrocnemius muscle (Figure 10B), quadriceps (Figure 10C), tibialis anterior muscle (Figure 10D) and soleus (Figure 10E), and epididymis fat mass (Figure 10F) were significantly reduced in LLC group. Luckily, we found that Aloin A (20 mg/kg) obviously recovered body weight and muscle mass reduction, but not epididymis fat mass in LLC tumor-bearing mice, suggesting that Aloin A could improve the cachexic condition of tumor-bearing mice.

### Effect of Aloin A on muscle histology in mice bearing lung tumor

**Figure 11:**
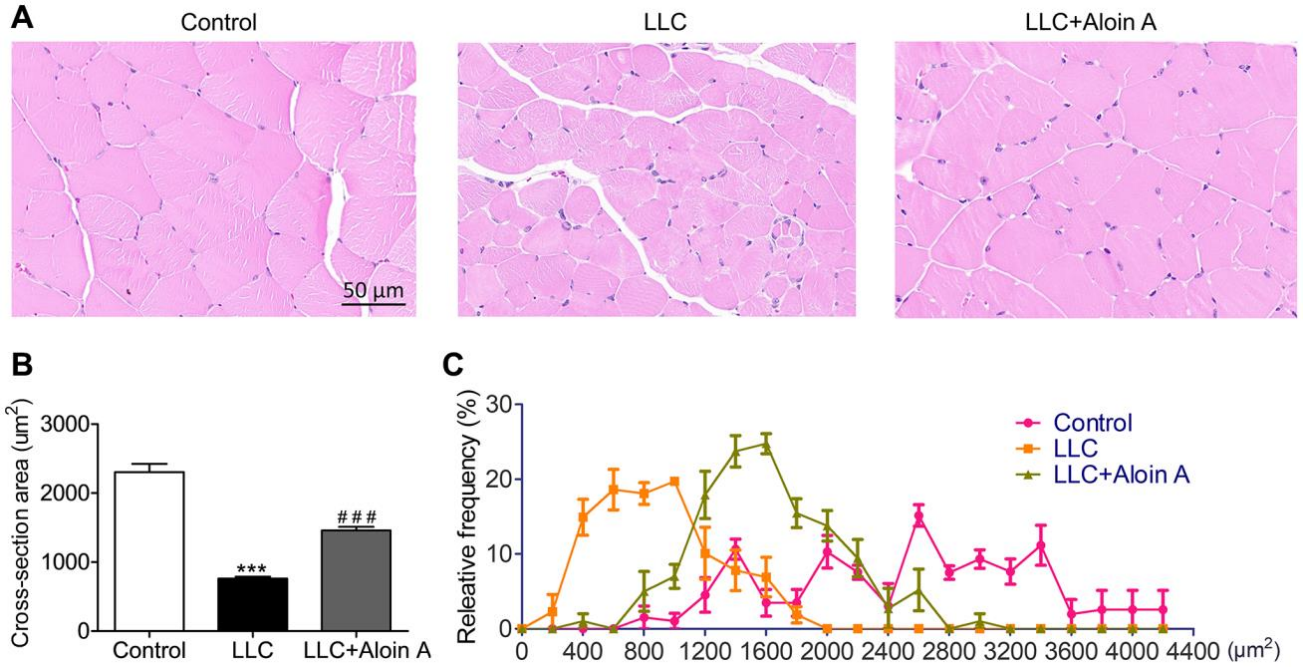
**Effect of Aloin A on muscle histology in mice bearing lung tumor.** (**A**) Representative images of hematoxylin and eosin (H&E) of transversal sections of the muscle. Scale bars: 50 μm. (**B**) The quantification of cross-sectional area (CSA) in muscle fibers. (**C**) Relative frequency of muscle fibers. The values are shown as the mean ± SEM (*n* = 3). ***P < 0.001 vs. the control group. ^###^*P* < 0.001 vs. the LLC group.

The results of HE staining revealed that compared with the control group, the cross-sectional area (CSA) of skeletal muscle in the LLC group was remarkably reduced (Figure 11A, 11B), and Aloin A (20 mg/kg) treatment obviously increased CAS in LLC tumor-bearing mice. Moreover, the muscle fibers in control group was more distributed between 1400-3400 μm2 while most of muscle fibers in LLC group are distributed between 200 and 1600 μm2. After treatment, we found that Aloin A increased the distribution and frequency of big fibers, most of them are between 1000 and 2600 μm2 in LLC tumor bearing mice (Figure 11C). These results further demonstrated that Aloin A can effectively rescue cachexia-induced muscle atrophy in tumor-bearing mice.

### Effect of Aloin A on mRNA expression of key genes in skeletal muscle of mice

**Figure 12:**
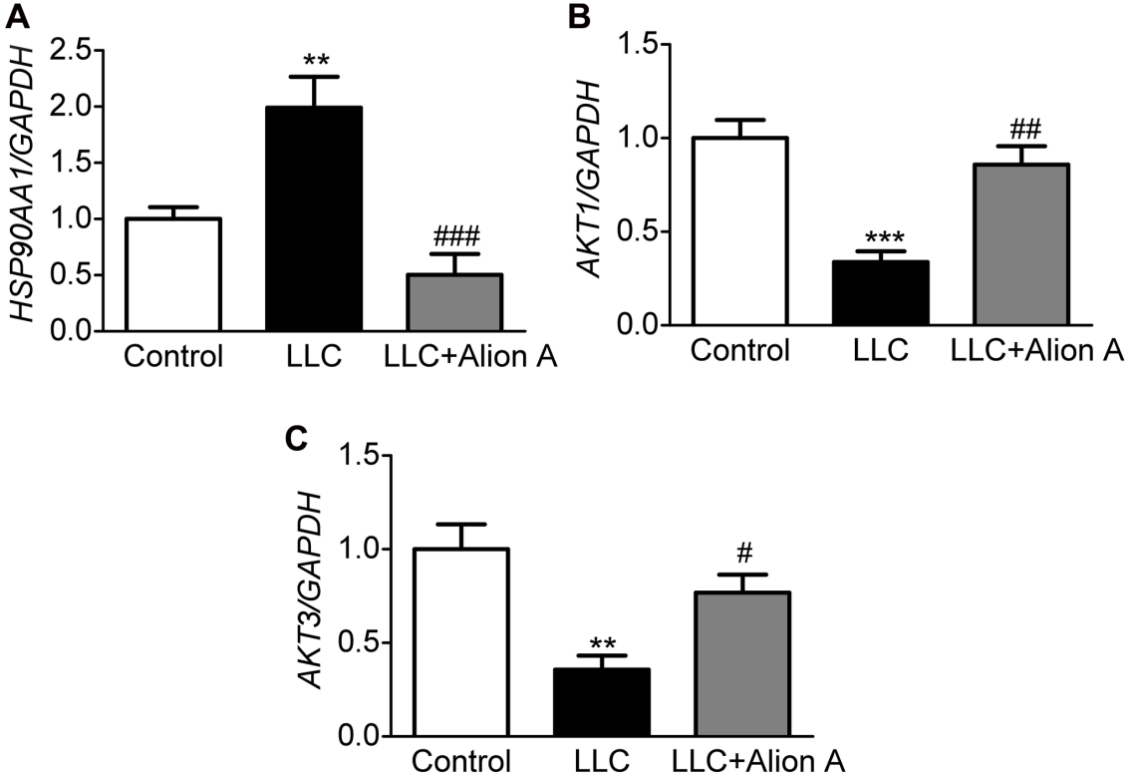
**Effect of Aloin A on mRNA expression of HSP90AA1, AKT1 and AKT3 and in skeletal muscle of mice.** (**A**) mRNA expression of HAP90AA1. (**B**) mRNA expression ratio of *AKT1*. (**C**) mRNA expression of *AKT3*. The values are shown as the mean ± SEM (*n* = 6). ^**^P < 0.01 vs. the control group. ^#^*P* < 0.05, ^##^*P* < 0.01, ^###^*P* < 0.001 vs. the LLC group.

Based on the results of systems pharmacology, molecular docking, and MD, HSP90AA1, AKT1, and AKT3 may be key genes involved in the anti-cachectic effect of Aloin A against muscle atrophy in tumor-bearing mice, and we further validated these hub targets through RT-qPCR, and found that Aloin A (20 mg/kg) treatment significantly down-regulated the mRNA expression of *HSP90AA1* (Figure 12A) while upregulated the mRNA expression of *AKT1* and *AKT3* (Figure 12B, 12C) in LLC tumor-bearing mice, suggesting that these targets are, indeed, contributing to muscle atrophy in tumor bearing mice, and Aloin A, at least in part, may alleviate the muscle atrophy via improving abnormal gene expression of *HSP90AA1, AKT1 and AKT3*.

## DISCUSSION

At present, there are no effective medical interventions and approved drugs to treat cancer cachexia, and adequate nutritional support is regarded as the main intervention [32]. In recent years, systems pharmacology-based strategy has become an important approach to investigating the relationship between drugs and diseases and drug discovery from TCM [33, 34]. In this study, we used network pharmacology, molecular docking, molecular dynamics and experimental validation to explore the pharmacological and molecular mechanisms of the anti-cachectic effect of Aloin A. Firstly, we used systems pharmacology estimated the targets of Aloin A and targets of CC-induced muscle atrophy by using several databases, then constructed PPI and predicted hub targets, and conducted Gene Ontology, KEGG pathway enrichment. Furthermore, the key targets were validated by molecular docking and molecular dynamics. Finally, we further investigated the anti-cachectic effect of Aloin A in mice bearing lung tumors.

It is well accepted that cancer cachexia is mainly induced by inflammation, oxidative stress, apoptosis, etc. [1], and Aloin A, a main bioactive component extracted from *Aloe vera*, has a wide range of pharmacological activities, including anti-inflammation, anti-tumor, anti-oxidative stress [13, 14]. These implications laid the foundation for exploring the efficacy and mechanism of Aloin A in relieving cancer cachexia. Based on network pharmacology, we assessed overlapping targets between Aloin A and CC-induced muscle atrophy. Among them, the core genes are HSP90AA1, AKT1, CTNNB1, BCL2L1, RELA, TNF, AKT3. In addition, we explored their GO-enriched functions and KEGG pathways, and found that these targets were closely related to inflammatory response, apoptosis, and smooth muscle cell proliferation, as well as protein degradation-related signaling pathways, such as AGE-RAGE, MAPK, Wnt signaling pathways, and protein synthesis-related pathway PI3K/Akt signaling, suggesting that they may be the main targets of Aloin A in alleviating CC-induced muscle atrophy.

It is well known that inflammation is one of the hallmarks of cachexia [17]. The role of TNF-α, the mediator of chronic inflammation, in muscle wasting has been well studied in both human and preclinical animal models [35]. It is reported that TNF-α could activate skeletal muscle nuclear factor κB (NF-κB) transcription factor and promote protein degradation [35, 36]. Recently, some researchers have found that a number of tumor cells express high levels of cell surface heat shock protein (HSP)90 that cause systemic inflammation, and induce myotube Atrophy [37] and muscle catabolism by activating STAT3 [38] and TLR4 [39] signaling in tumor cachexia models. Moreover, inhibition of HSP90 could significantly ameliorate muscle wasting in cancer cachexia mice [21, 38]. In this study, we used network pharmacology to predicted hub genes, and PPI results showed that inflammation related genes, including TNF, RELA, namely p65-NF-ΚB, and HSP90AA1, namely HSP90, are the hub targets, and KEGG pathway enrichment analysis revealed that the inflammation related TNF, NF-κB, TLR4 and STAT3 signaling pathway involved in the effect of Aloin A against CC-induced muscle atrophy.

It is reported that PI3K-Akt signaling pathway is one of the important intracellular pathways regulating adult skeletal muscle function and atrophy [40, 41], and AKT is a key intermediate in insulin/IGF-1-mediated muscle wasting [42]. Consistent with this research, AKT activity is reduced in several models of muscle atrophy, including sarcopenia and diabetes [43, 44]. Recently, researchers have found that activation of AKT decreases protein degradation and increases protein synthesis, leading to the growth of adult skeletal muscle [45, 46]. Moreover, activation of AKT could inhibit apoptosis through FOXO3 [47], and apoptosis signaling precedes protein degradation in wasting skeletal muscle during catabolic conditions [48, 49]. Apoptosis markers were observed in severely cachectic muscle of Apc (Min/+) mice [50]. Recently, it has been reported that cancer might induce muscle atrophy by targeting Bcl-2-mediated apoptosis and overexpression of Bcl-2 successfully reversed atrophy of C2C12 myoblasts [51]. In this study, our network pharmacology results showed that AKT1, AKT3, and BCL2 are hub targets, and KEGG pathway enrichment analysis revealed that PI3K-AKT signaling pathway is involved in the effect of Aloin A against cancer-related muscle atrophy.

To validate the network pharmacology results and better understand protein-ligand interactions, we applied molecular docking and molecular dynamics (MD) simulation. Molecular docking is a structure-based method that enables the identification of novel compounds of therapeutic targets by predicting ligand–target interactions at a molecular level [52, 53]. The more negative the binding energy, the more stable the binding of the compound to the target [54]. Our molecular docking results showed that Aloin A had good binding interactions with HSP90AA1, AKT1, CTNNB1, BCL2L1, RELA, TNF, AKT3, the ranging from −7.9 to −8.9 kcal/mol, suggesting that Aloin A stably combined with these proteins for attenuating cancer related muscle atrophy. Molecular dynamics (MD) simulation is important in comprehending conformational changes and dynamic mechanisms of proteins and is commonly employed in drug design [55] and target validation. This technology could simulate different experimental conditions, such as changes in temperature, pH, and solvent. Therefore, we further validated the key targets, such as HSP90AA1, AKT1, AKT3, with MD simulation, and found that the RMSD, RMSF, Rg and binding free energies trajectory of the HSP90AA1-Aloin A, AKT1-Aloin A and AKT3-Aloin A complexes became stable, but among the three complexes, the stability of the HSP90AA1-Aloin A complex is the best. These above results indicate that after the small molecule ligand combines with this protein, the combination is relatively stable and reliable.

Although computational methods, including network pharmacology, molecular docking, MD, etc., are a useful tool and strategy to predict underlying therapeutic mechanisms and provide research directions [56], given the possible spurious relations between one database and another, the combination of computer simulations and traditional experimental methods is an ineluctable requirement for the development of drug discovery to deepen understanding of the interaction mechanisms of small ingredients and proteins [57]. Therefore, we applied animal experiments to further validate the effect of Aloin A against CC-induced muscle atrophy, and confirmed the predicted targets by gene expression. Cancer cachexia is a multifactorial syndrome characterized by muscle weakness, loss of body weight, muscle wasting, and adipose tissue depletion during cancer development [1]. Body weight loss and associated muscle wasting lead to progressive impairment of muscle function [58]. In animal models, muscle function is measured by grip strength test [58], inverted screen test [59, 60], rotarod test [61], which can be used as reliable markers to estimate functional muscle status. In this study, we found that Aloin A ameliorates muscle function by increasing muscle strength, including the improvement of rotarod time, hanging time, and frailty score, in cachexia mice. Moreover, we further studied the effect on loss of body weight and muscle mass, and found that Aloin A not only attenuated the loss of body weight and skeletal muscle mass, including gastrocnemius muscle, quadriceps, tibialis anterior and soleus, but also improved mean CSA and distribution of muscle fibers, suggesting that Aloin A could effectively ameliorate muscle atrophy and strength in cancer cachexia. Moreover, we performed RT-PCR for the validation of predicted targets, and found that Aloin A could effectively improve abnormal gene expression of HSP90AA1, AKT1, and AKT3 in skeletal muscle of cachexic mice bearing tumors, suggesting that Aloin A, at least in part, could attenuate CC-induced muscle atrophy by mediating HSP90AA1/AKT signaling. However, we have to acknowledge some limitation of this study. We have not used SPR, relevant inhibitors or the conditional knockout mice to validate the effect of Aloin A with specific targets, which should be studied in the future.

## CONCLUSION

In summary, our research systematically investigated Aloin A from a whole action mechanism perspective in the treatment of CC-induced muscle atrophy. We successfully discovered the potential role, mechanism and key targets of Aloin A against CC-induced muscle atrophy by using network pharmacology, molecular docking, molecular dynamics and experimental validation, and found that Aloin A realizes the therapeutic effect of cancer cachexia through multiple targets and pathways, including HSP90AA1/AKT signaling, which provides evidence for Aloin A as a potential drug for the treatment of cancer cachexia in a clinic.
